# Sublethal costs of infection reduce performance of collective tasks in social insects: a modelling study

**DOI:** 10.1038/s44434-026-00007-z

**Published:** 2026-11-05

**Authors:** Supraja Rajagopal, Takao Sasaki, Richard J. Hall

**Affiliations:** 1 Odum School of Ecology, University of Georgia, Athens, GA USA; 2 Center for the Ecology of Infectious Diseases, University of Georgia, Athens, GA USA; 3 Brain and Cognitive Sciences, University of Rochester, Rochester, NY USA; 4 Department of Infectious Diseases, University of Georgia, Athens, GA USA

**Keywords:** Ecology, Ecology, Zoology

## Abstract

Group living confers benefits such as improved foraging efficiency, but social insects also face costs, including increased risk of parasitism. Infection can alter individual and social behaviours, potentially reducing the performance of collective tasks through impaired individual performance, reduced recruitment, and disrupted information transfer. Alternatively, interactions with uninfected groupmates may buffer these effects by facilitating social learning. However, we lack a mechanistic framework for quantifying how infection influences collective behaviours through direct and socially mediated effects. We developed an agent-based model, parameterized for the ant *Temnothorax rugatulus*, to investigate how infection influences collective foraging. We varied the magnitude of behavioural costs and infection prevalence and recorded effects on colony performance and reciprocal effects of infected individuals on uninfected individuals (and vice-versa). Individual-level behavioural costs had the greatest impact on collective performance when they occurred early in the foraging process, while reduced recruitment further amplified these effects. We also found reciprocal effects between infected and uninfected ants that scale with infection prevalence, suggesting social interactions can buffer declines in colony performance only when infection levels are low. Our study provides a mechanistic understanding of how infection-related changes in behaviour can impact groups, with important consequences for fitness of group-living insects.

## Introduction

Social living is widespread across the animal kingdom, including social insects that perform critical ecosystem services. Group living has evolved across species as sociality confers many benefits, including anti-predator defenses and higher efficiency in tasks like brood care and foraging^1,2^. However, group living can also result in costs due to increased risk of transmission of pathogens among group members^3–7^. Pathogen transmission is often higher in social insects due to increased density of animals, limited dispersal opportunities and low genetic diversity within colonies which can increase susceptibility to shared pathogens^8,9^. However, social insects have evolved collective behavioural, organisational and physiological defenses against parasitism, known as social immunity^10–14^. Nevertheless, sublethal effects of pathogens on individual behaviours can scale up and affect inter-individual interactions in the group. Since these local interactions underpin collective tasks essential for colony survival like foraging, nest construction, and defense; pathogen-induced behavioural changes can have profound consequences for colony functioning^15^. Given the vital ecosystem services provided by social insects, such as pollination, soil turnover, and nutrient cycling, understanding how infection affects their collective performance is critically important^16,17^.

Infected individuals often exhibit sickness behaviours in response to infection, including reduced activity levels that allow them to direct energy towards immune responses and recuperation^18^. Sick individuals may walk slower, get exhausted sooner, show lower ability to sense food cues, or take inefficient routes when walking compared to healthy individuals (Table 1). For example, red colobus monkeys rest more when infected with whipworm and *Nosema* infected honeybees show increased hunger cues^12,19^. For group living animals, these sickness behaviours can be modulated by the social context, enabling them to forgo sickness behaviours when necessary (for example, ants in a starving colony may still go out to forage)^18,20^. Social animals may avoid or alter their interactions with infected individuals and vice versa. In *Lasius niger* ants and wild house mice, immune-challenged individuals actively avoided conspecifics^21,22^.

**Table 1:** Behaviours relevant to foraging that could be altered by infection in individuals and examples from real host–pathogen systems

Behaviour affected	Model implementation	Parameter affected	Examples
Locomotion	Lowered walk speed	walk_speed	Honey bees (*A. mellifera*) injected with a sham immune challenge (LPS) show reduced locomotor activity^45^
Food sensing	Radius at which ants perceive food is reduced	d_food	Fruit flies (*D. melanogaster*) infected with *Wolbachia* bacteria show lower responsiveness to food cues^46^
Food sensing	Sinuosity of path once food is perceived is higher	α_food	Ants (*C. floridanus*) infected with *Ophiocordyceps* fungi make erratic and undirected foraging trips^47^
Social	Probability of participating in a tandem run is reduced	p_leading, p_following	Ants (*C. aethiops*) infected by *Metarhizium brunneum* fungi reduced social interactions with healthy nestmates^48^ *A. mellifera* injected with LPS spend less time on social behaviours^45^
Endurance	Time individuals spend scouting unsuccessful is higher	giving_up_time	Allenby’s gerbils (*Gerbillus andersoni allenbyi)* parasitized by fleas (*Synosternus cleopatrae pyramidis*) spend less time in resource patches when foraging^49^

Collective outcomes emerge from inter-individual interactions through the sharing of information among group members^15,23^. Sublethal costs of infection to individual behaviour can affect this information sharing in two ways - by affecting the ability of group members to participate in information sharing, or by affecting the quality of information shared^24^. Infected individuals may be less active or interact less with susceptible group members, leading to reduced number of participants in a collective task. For example, fire ants infected with SINV-1 show reduced recruitment efficiency when foraging, which decreased the colony’s foraging intensity^25^. If infection does not alter social interactions but affects other aspects of individual behaviour, information exchange may still be impaired, leading to reduced collective performance. For instance, pairs of sticklebacks infected with *Schistocephalus solidus* show reduced alignment and cohesion even when only one individual is infected^26^. Alternatively, if infected individuals gain reliable information from non-infected group members, social interactions may mitigate individual-level costs of infection and buffer its impacts on collective performance. For example, shoaling sticklebacks can overcome costs to individual infection at the group level if uninfected individuals outnumber infected individuals^27^. The impact of pathogens on collective outcomes depends on the balance between information loss (via reduced participation or reliability) and information compensation (via social buffering), as well as infection prevalence, colony demands, and the redundancy of information within the group^24,28^. Although infection is known to alter individual behaviour and social interactions, how pathogen-induced changes in participation and information quality scale up to influence collective outcomes remains poorly understood.

**Fig. 1:**
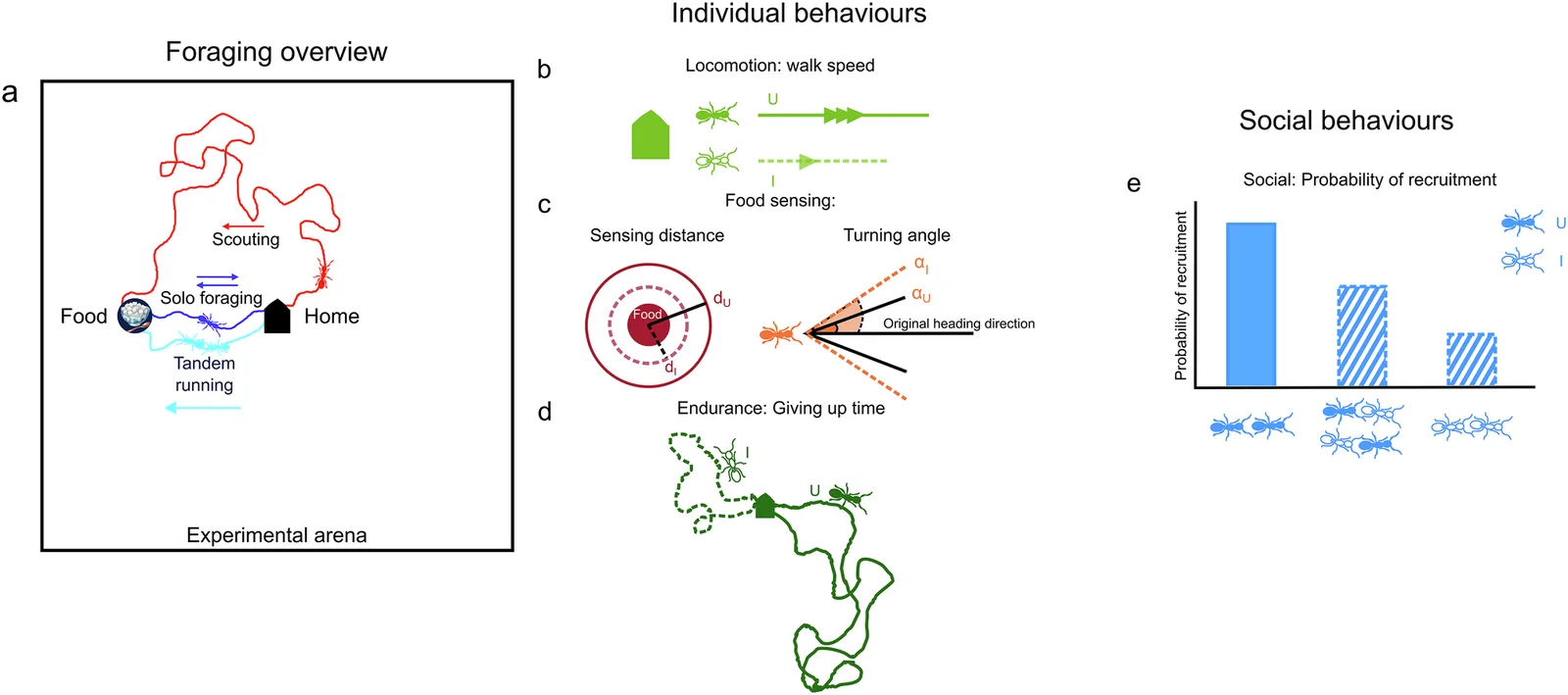
Overview of simulated experimental setup and behaviours impacted by infection in model. **a** Simulated arena and representative tracks of the routes made by the agents. **b**–**e** Visualization of how different sublethal costs of infection in Table 1 influence foraging ants: **b** infection reduces walk speed, and thus the distance walked in one time step; **c** infection reduces food sensory ability: infected ants must be closer to the food to detect it (red) or infected ants orient less well towards the food after infection (i.e. they have a wider turning angle than uninfected ants, orange); **d** infection reduces endurance, so that infected ants give up searching for food sooner (and thus move less far) than uninfected ants; **e** infection reduces recruitment to the collective task according to the infection status (U uninfected, I infected) of the initiator and potential recruitee, so that U-I pairs form less frequently than U-U pairs, and I-I pairs form less frequently than U-I pairs.

We developed an agent-based model to explore how sublethal costs of infection to different individual behaviours, and social interactions, affect collective foraging outcomes in a social insect, applied to the rock ant *Temnothorax rugatulus*. Collective foraging behaviour in *Temnothorax* ants has been studied extensively, making it an ideal system to study with theoretical models. These ants live in colonies of 70–200 workers, with our simulated colonies having 100 workers^29^. During collective foraging, scout ants (25% of colony^17^)leave the nest in search of food, walking randomly until they sense the food, when they move more directly to the food. When scouting ants find food, they can make repeat visits to the learned food site or recruit other individuals (Fig. 1a) (see Methods).

In our model, we varied the magnitude of the impact of infection on different processes relating to individual movement capacity, sensory cues, and dyadic interactions relating to recruitment to the foraging task (Table 1, Fig. 1b–e). The cost of infection to individuals was modelled as a proportional reduction in a model parameter (e.g., walking speed) relative to an uninfected individual. It ranges from 0 (no cost) to 1 (failure to perform the behaviour). We assessed impacts of infection on colony-level performance by quantifying the net energy gain to the colony, which we calculate as the energy gained by bringing food to the colony minus the total energy expenditure of all ants engaged in foraging. We then normalized this relative to the performance on uninfected colonies so that a relative colony performance of 100% would correspond to no impacts of infection on colony performance. To understand if infected and uninfected ants influence each other’s performance, we first measured the average performance of an uninfected and infected individual as the net energy brought to the colony by ants of this infection status, divided by the number of ants of this infection status. We calculated these performance metrics under different costs of infection and colony-level infection prevalences, and assessed whether reciprocal effects of infection occurred by comparing individual performance of each infection status to performance of colony where all individuals had the same infection status (i.e. how much did uninfected ant performance drop in the presence of infection relative to performance in an uninfected colony, and how much did infected ant performance increase in the presence of uninfected conspecifics relative to a colony where 100% of ants were infected). Additional details of these metrics are provided in the Methods section.

We predicted that (i) colony-level impacts of infection on collective performance would increase with increasing infection costs and infection prevalence; (ii) impacts on colony performance would differ according to the individual behaviour impacted by infection; and (iii) costs of infection to social processes would exacerbate effects of individual costs of infection to further reduce performance. We also predicted that uninfected and infected hosts would have reciprocal effects on each other’s performance. Specifically, we predicted that over the duration of a collective task, the average performance of individuals of one infection status (uninfected or infected) would be contingent on the relative frequency of individuals of the other infection status, because this would determine their interaction frequency, and thus the probability of recruitment to a collective task.

## Results

### Effects of infection on colony-level foraging performance

**Fig. 2:**
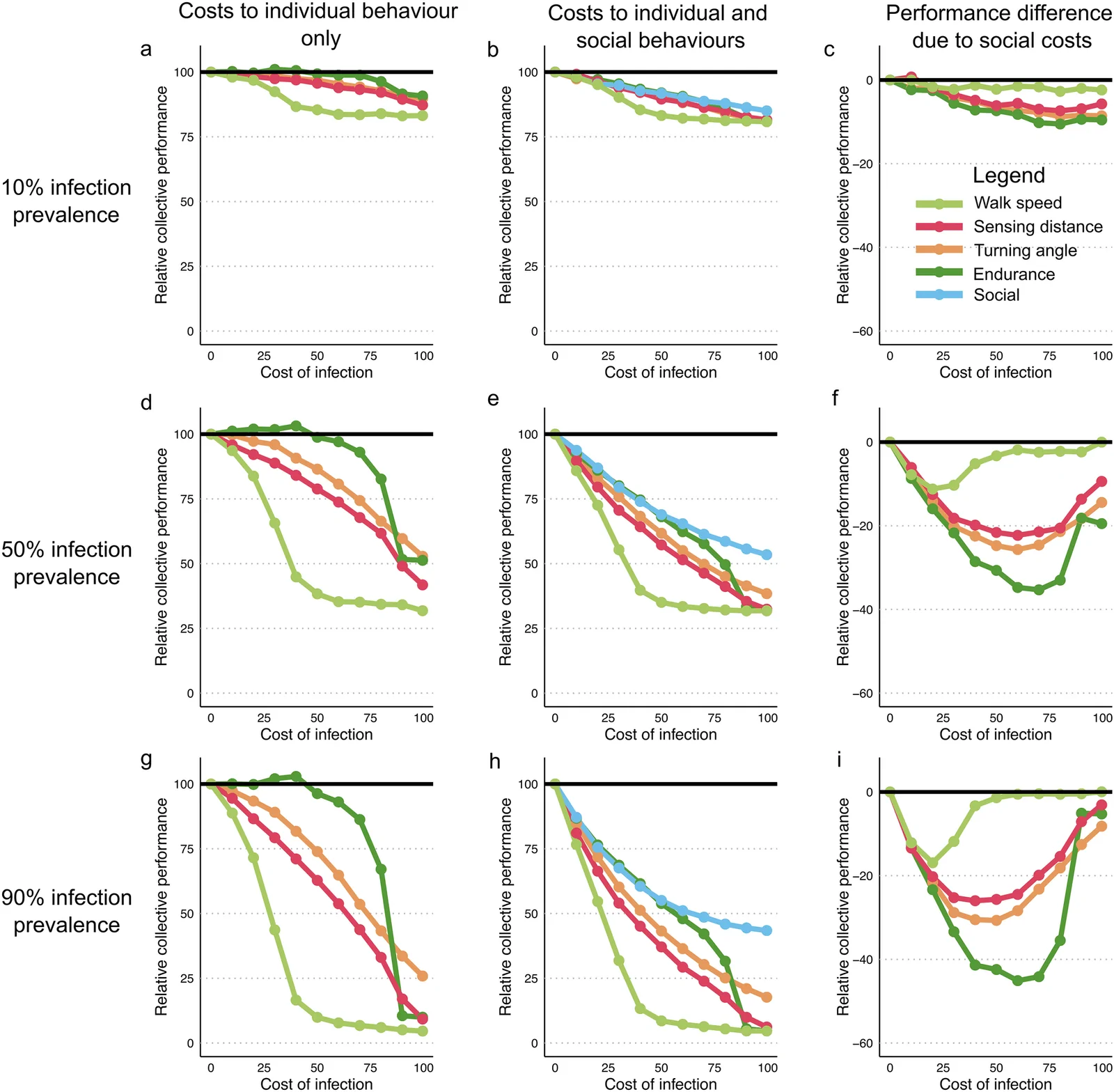
Relative colony performance as a function of infection costs across varying levels of infection prevalence. Rows indicate the level of infection in the colony – prevalence can be low (**a**–**c**; 10%,), medium (**d**–**f**; 50%) or high (g,h,i; 90%). **a**–**g** Relative colony performance when individual behaviours are impacted by infection. Colours indicate which individual behaviours are impacted by infection (light green - walk speed, red - distance at which food cue is perceived, orange - turning angle, dark green - giving up time). **b**, **e**, **h** Relative colony performance when both individual and social costs of infection occur, the blue line is the cost of infection to social interactions only (i.e. reduced probability of recruitment when at least one individual in the dyad is infected), and the other lines indicate colony performance when this social cost of infection is combined with a cost of infection to each of the individual behaviours (same colour scheme as the first column) Relative colony performance is the net energy gain to an infected colony during one 90 min foraging bout (food brought to the colony minus the total energy expenditure of all ants engaged in the foraging task) normalized by the performance of an infection free colony; and the cost of infection is the proportional reduction in an individual or social behaviour due to infection (see Methods). Each of these metrics is multiplied by 100 so that results are expressed as a percentage. **c**, **f**, **i** The difference in relative colony performance due to social costs of infection, calculated by subtracting the performance of its coloured line in the second column (**b**, **e**, **h**) from the corresponding line in the first column (**a**, **d**, **g**). In all figures the black horizontal line represents the performance of a disease-free colony and plotted values represent the mean performance averaged over 1000 simulation runs

For each of the individual behaviours that could be impacted by infection (walk speed, food-sensing distance and orientation towards food, and endurance), colony performance (i.e., the net energy gain from foraging) decreases as the magnitude of the cost of infection to each behavioural process increased, and when a higher proportion of the colony is infected (Fig. 2a, d, g). Costs to infection are more detrimental to collective performance when infection impacts behaviours that occur earlier in the foraging process. This is because the earlier individual behaviour is impaired, the longer it takes to return to the nest and recruit other individuals, reducing the benefit of information sharing to the colony. For example, reduced walk speed impacts infected ants from the onset of foraging, increasing time to food detection while reduced sensing and orientation towards food increases time to reach the detected food, return to the nest and subsequently recruit other individuals. As further evidence that the ordering of tasks drives how infection affects collective outcomes, the number of recruitment events plotted as a function of costs of infection follows the same patterns as colony performance (Supplementary Fig. S2a). The impact of reduced endurance (i.e. infected ants give up searching for food more quickly than uninfected ants) on colony performance is negligible at low costs of infection until a threshold cost of infection, after which colony performance drops sharply (Fig. 2d, g). This is because at high costs of infection, most infected scouts give up before encountering food and thus fail to recruit additional ants to the foraging task.

When infection only impacts social behaviour via reduced recruitment success, the effects on colony performance are smaller in magnitude than similarly-sized costs of infection to individual behaviours, because they occur later in the foraging process, and saturate at high costs of infection, when recruitment only occurs between uninfected-uninfected pairs (Fig. 2b, e, h blue line). When infected individuals experienced impacts to both individual performance and reduced recruitment success, the colony-level performance dropped more sharply than when only individual behaviour is impacted by infection, but the pattern of infection impacts on behaviours occurring earlier in the foraging process being more detrimental to collective performance still held (Fig. 2b, e, h). The drop in performance due to co-varying social costs was the highest at intermediate costs of infection (Fig. 2c, f, i). This is because high costs to individual behaviours delay or prevent recruitment, reducing the relative impact of social costs.

Across the individual and social costs of infection explored, the magnitude of declines in colony performance increases with increasing infection prevalence, but qualitative patterns of decline and relative magnitude of effects among the behavioural processes impacted remain consistent across prevalence levels (Fig. 2, Supplementary Fig. S1). At low infection prevalence (10%) colonies can buffer infection costs to individuals - at the highest cost of infection, colonies experience up to a 10% drop in collective performance, and co-varying individual and social costs does not lower collective performance (Fig. 2a–c). The qualitative patterns of colony performance decline and relative magnitude of these declines across behavioural processes are robust to alternative assumptions about how tandem pairs experience costs of infection (i.e. whether the infection impacts the movement of the tandem pair only if the leader is infected, as assumed in our main results, or whether either an infected leader or follower determines the tandem movement, Supplementary Fig. S3); colony performance is slightly lower when tandem performance is impacted by an infected leader or follower than when performance is only impacted by the leader being infected. However, when either an infected leader or follower determines the tandem movement, we see that colony performance is lower when only walk speed is impacted compared to when walk speed and recruitment are affected by infection (Supplementary Fig. S3). Our principal findings that ordering of behavioural processes determines performance declines with infection, and compounding of costs when both social and individual costs are impacted, are also robust to realistic variation in our baseline infection-free parameters (Supplementary Figs. S5, S6); quantitatively, the magnitudes of performance declines with infection are most sensitive to variation in walk speed.

### Reciprocal effects of uninfected and infected individuals on each other’s performance

**Fig. 3:**
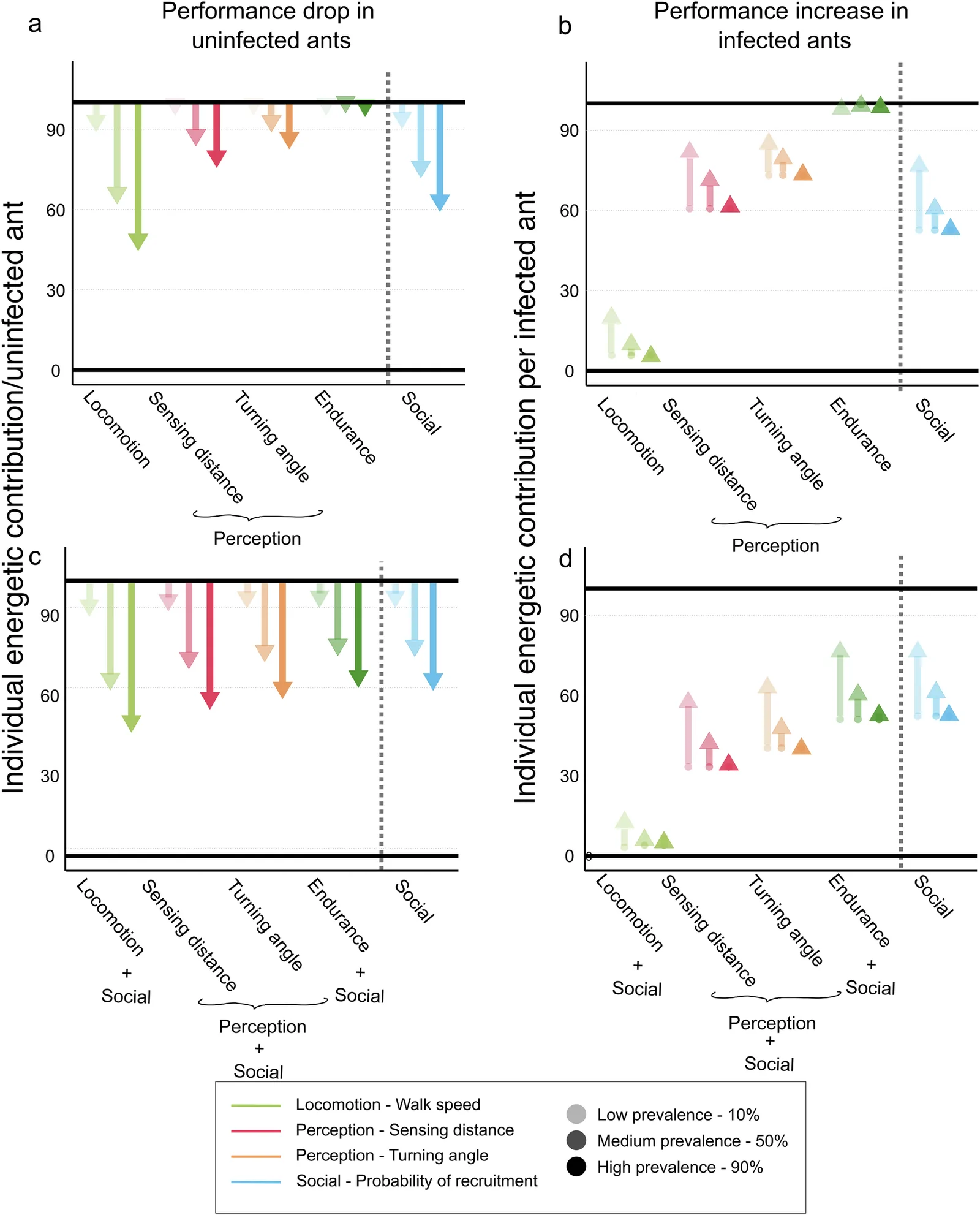
Reciprocal effects of infection status on individual performance (i.e. the average energetic contribution of individuals according to their infection status). Columns indicate (left) the reduction in uninfected individuals’ performance due to infected individuals and (right) the increase in infected individuals’ performance through interactions with uninfected individuals; rows indicate (top) infection costs to individual behaviours only (indicated by arrow colour) or (bottom) infection costs to both individual and social behaviour. **a** On average, relative energetic contribution of uninfected individuals decreases with increasing infection prevalence (indicated by arrow opacity) and **b** relative energetic contribution of infected individuals increases when uninfected individuals are relatively common. Reciprocal effects of infection status on individual performance when explicitly co-varying individual costs with social costs; **c** on average, relative energetic contribution of uninfected individuals decreases with increasing infection prevalence and **d** relative energetic contribution of infected individuals increases when uninfected individuals are relatively common. Relative energetic contribution of an individual is the net energy gained by an ant (food brought minus energy expended by walking) normalized by the average energy contribution of an uninfected ant (see Methods). This metric is multiplied by 100 so that results are expressed as a percentage. Line colours represent the behaviour impacted by infection, line opacity indicates the infection prevalence, and the cost of infection to the impacted behaviour is 50%. The dots at the base of each vertical arrow represent performance when all individuals have the same infection status (all uninfected in (**a**, **c**), all infected in (**b**, **d**)), and the arrowheads represent the average performance of individuals of one infection status in the presence of individuals of the opposite infection status. Plotted values represent the average individual performance over 100 simulation runs.

Our results show that the average performance of uninfected or infected individuals is affected by the individuals with the opposite infection status, and that the magnitude of the performance change depends on the infection prevalence (Fig. 3a, b). Infected individuals caused an ~40% reduction in performance of uninfected individuals when infection prevalence was high, and when infection impacted walk speed or recruitment probability. Uninfected individuals boosted the performance of infected individuals by ~30% when infection prevalence was low, and when infection reduced recruitment probability. When infection affected social costs in addition to individual behavioural costs, we found that the magnitude of these reciprocal impacts increased for all behaviours (Fig. 3c, d). The average performance of infected individuals increases by ~30% at low (10%) infection prevalence (Fig. 3d). Under alternative assumptions about how tandem pairs experience costs of infection; impacts of infected individuals on uninfected individuals are stronger, with an increased drop in the average performance of uninfected ants when infection impacts the movement of tandem pair when either leader or follower are infected (Supplementary Fig. S4). In this scenario, the improvement in average performance of infected individuals is lower, as any recruitment route is impacted by infection (Supplementary Fig. S4).

## Discussion

Understanding the interplay between collective behaviour and infectious disease is essential to advancing both fields, as host behaviour and parasite infection are reciprocal processes that drive each other^28,30,31^. In social insects, infection in colonies can be devastating as changes in individual behaviours could affect information sharing among group members, affecting their ability to carry out collective tasks critical to colony survival like foraging, nest searching and defense. Our study investigated how sublethal infection-induced behavioural changes in individuals and social interactions can modulate colony-level outcomes. We highlight two important mechanisms by which individual changes in behaviour can modulate colony-level outcomes - through ordering of behaviours in a collective task and through social interactions during recruitment. Costs to individual behaviour had a stronger impact on collective outcomes when infection affected behaviours that take place earlier in the collective task. These colony-level effects were exacerbated when individual behaviours and social interactions were both affected by infection. We also found reciprocal effects of uninfected and infected individuals on each other’s performance. The magnitude of these effects was exacerbated when infection reduced social recruitment to the task.

Our simulation study suggests that sublethal costs of infection to individuals can substantially reduce collective performance by directly impacting individual performance, and indirectly via interactions with infected individuals. Our study is based on collective foraging behaviours of *T. rugatulus* colonies, but these mechanisms by which individual costs affect groups may apply more generally across group activities and taxa where information sharing is decentralized, like nest emigration in social insect colonies and migration in locust swarms^32,33^. In these social groups, suboptimal performance by any one individual propagates through the group through information sharing, affecting collective outcomes. Our model results suggest that in decentralized systems, the order of behaviours in the collective process is a primary driver of how infection of individuals affects collective outcomes. When behaviours that take place early in the collective task are affected by infection, this slows down feedback to the collective task, as recruitment cannot take place without knowledgeable individuals in the group. These effects are mediated by interactions among individuals in the group where they share information about the collective task and invite conspecifics to join the collective task.

Parasites can alter host social networks by affecting how likely susceptible and infected individuals are to interact with each other. Our study found reciprocal effects of individuals of opposite infection status on each other’s performance, contingent on how much infection reduces the probability of recruiting (or being recruited). The strength of these reciprocal impacts, and if they are a net positive or negative at the colony level, may depend on the infection status of the information provider versus the information receiver. In insects with direct recruitment mechanisms, like *Temnothorax rugatulus*, infected ants that are unable to independently navigate to a target may still be able to follow a knowledgeable uninfected leader. In other tasks, such as nest relocation, ants may be carried from the old to the new nest, which would also circumvent costs to movement capacity or navigation of an infected recruit. On the other hand, being recruited by an infected individual could lead to the propagation of low-quality information among colony members - for example, honeybees with IAPV and DWV show impaired sensory perception and learning, which could lead to infected bees recruiting foragers to sub-optimal food sources^14,34^. These differences may explain why infection prevalence determined the average effect on individual performance: when most information providers are uninfected (i.e. infection prevalence is low), they can buffer infection impacts on the colony, while if information providers are more likely to be infected (i.e. prevalence is high), the transfer of poor-quality information can lead to compounding impacts on colony outcomes.

Social animals use a wide range of behaviours to recruit group members, and the nature of recruitment mechanisms can influence the robustness of the patterns of collective outcomes we observed. Our model is based on a system with direct one-to-one recruitment strategies with decentralized control. In systems with centralized control (for e.g. flocks of homing pigeons^35^), infection-induced changes to the behaviour of highly influential and central individuals could have a disproportionally large impact on collective outcomes, causing sharp declines in colony performance when these individuals are infected^24^. Some group living animals use one-to-many recruitment mechanisms, such as pheromone trail laying ants^16^ where one knowledgeable individual recruits many naive individuals. Here, recruitment rapidly amplifies participation of the group and increases the speed of information transfer^34,36^, and infection-induced changes to recruitment behaviour may have highly deleterious consequences for the group. Our model shows that even in animals using one-to-one recruitment, missed recruitment opportunities and knock-on effects of individual infection can lead to colony-level reduction in performance. In different recruitment systems where one individual can recruit multiple naive individuals, we would expect to see stronger reciprocal effects.

In our model, we measure colony-level benefits and costs over one short foraging bout, ignoring changes in population size and infection prevalence due to transmission and disease-induced mortality that could exacerbate infection effects on collective behaviour beyond the sub-lethal effects we explored. Collective behaviour and parasite infection can be linked by feedback, where infection affects the collective task and spread of information, and these changes to the collective task further influence disease spread. For example, in swarming locusts, aggregation can increase parasitic transmission, but infection downregulates swarming behaviour, reducing transmission^33^. Infection-collective behaviour feedback may be contingent on the mode of parasite transmission. If participation in a collective task exposes individuals to disease vectors, recruitment mechanisms that lead to rapid increases in participation of individuals could have a beneficial effect of reducing transmission through encounter dilution^5,37^. In these systems, these feedbacks can buffer the impact of infection on performance of collective tasks and act as a form of social immunity^38^. Our results suggest that at low infection prevalence, social immunity has minimal impacts on task performance, but at high prevalence, avoidance of social contacts that could lead to transmission likely trades off with reductions in collective performance. When social recruitment is coupled to transmission risk (e.g. for contact-transmitted pathogens), initially infection spread through the population is facilitated by recruitment events. However, if infection costs decrease the ability of infected individuals to recruit, this could slow subsequent infection spread, regulating disease transmission but affecting the colony’s ability to perform collective tasks.

Disease-induced mortality could have differing impacts on collective outcomes dependent on whether lethal and sub-lethal effects of infection are correlated: for virulent pathogens with limited effects on host behaviour, early losses of hosts initiating a collective task could have cascading negative effects on colony performance through lost recruitment opportunities; alternatively, the culling of infected individuals with compromised behaviours could improve colony performance by removing knock-on impacts to social learning. Group living animals may face trade-offs between sharing beneficial information and transmission of parasites^39,40^ and may face selection to optimize social structures that balance the benefits of information sharing with costs of parasite transmission. This can depend on the life history and behaviour of the pathogen and host (e.g. pathogen virulence and mode of transmission, and information transfer in host). Highly virulent pathogens and novel emerging pathogens could have a big effect on this infection-information tradeoff, rendering species that rely on information sharing particularly vulnerable. Incorporating these effects and understanding group performance over longer timescales can also help understand the selection pressures on parasites and hosts and the feedbacks between them.

Collective animal behaviour and parasite transmission are often studied separately, despite being interrelated processes with reciprocal effects^40^. We currently lack an understanding of how and when infection-induced behavioural costs scale up to affect collective outcomes. Our study develops a simulation framework to understand how these infection costs create behavioural changes at the group level. We find that parasite infection to individuals disrupt inter-individual interactions, affecting collective performance. Future computational studies should study changes in population size and transmission over longer timescales, which could result in interesting feedbacks between collective behaviour and infection. Future studies should test our model predictions through lab and field studies in empirical systems. Social insects provide a tractable model system to study impacts to collective behaviour, as whole colonies can be collected and studied with relatively little effort^32^. In these systems, individuals can be administered immune challenges, and collective outcomes can be observed in controlled environments. Testing our model predictions across a broad range of host and parasite taxa will add to our understanding of the bidirectional connections between parasites and collective animal behaviour.

## Methods

### Approach and study system

To explore how different sub-lethal costs of infection influence collective performance in group-living animals, we developed an agent-based model (ABM). With agent-based modelling, researchers can program the behavioural rules of individual agents (in our model, ants) - how they interact with each other and their environment. ABMs are an ideal approach to understand decentralized systems, where local interactions generate system level outcomes. Using ABMs allow us to analyse how and why collective outcomes emerge, and are particularly useful in systems where agents are not identical^41^.

We used collective foraging in the ant *Temnothorax rugatulus* as our model system, as collective behaviour has been well studied in this group, allowing us to parameterize the model using empirical data (see Supplementary Material for more details). During foraging bouts, a small number of scout ants leave the nest in search of food. When the ants are close enough to food to sense it, they orient towards it. After finding food they return to the colony, where they can activate nest-mates to participate in the foraging task and lead them to the food (“tandem running”). These recruited ants learn more direct routes to the food, reducing their energy expenditure during foraging, and they may in turn recruit nest-mates, a positive feedback that amplifies the number of individuals participating. Together, these increase the efficiency of the collective task (the total amount of food acquired during a foraging bout relative to the total energy expended by foragers). While tandem running is a recruitment strategy specific to some ant species, the general principles of recruitment and positive feedback to the task broadly apply to many group-living animals.

In our agent-based model we allow infection to reduce the performance of infected individuals through costs to their movement capacity, their ability to detect and orient towards food, and their likelihood of sharing social information (Table 1). We explore how these different costs of infection scale up to influence collective performance. Below, we give an overview of how the ABM works; a more detailed description of the ABM following the ODD protocol^42^ is given in Supplementary Materials.

### Model setup

Our model was coded in the multi-agent programming language and modelling environment NETLOGO^43^. The model structure follows a prior ABM describing collective ant foraging^44^, which we modified to incorporate infection in ants.

Each simulation experiment has an ant colony at the centre of a virtual “experimental arena” of 140 cm × 140 cm, with a high-quality food source 40 cm away from the colony (Fig. 1a). This mimics lab-based experimental setups used to study collective behaviour in this ant species^43^ that inform model parameterization. An agent refers to an individual ant, which can move freely around the arena. Each simulation run has 100 agents, and 25% of the individuals are scout ants that independently venture outside the home nest at the start of each simulation. The rest of the individuals are potential recruits that leave the home nest only if recruited by another agent. The proportion of initial scout ants that independently forage is based on empirical observations on social insects^17^. The model updates every “tick”, which is equivalent to 1 second, and each simulation runs for 5400 time steps (equivalent to 90 min), based on empirical observations of the duration of collective foraging in this ant species^43^.

### Ant foraging

**Fig. 4:**
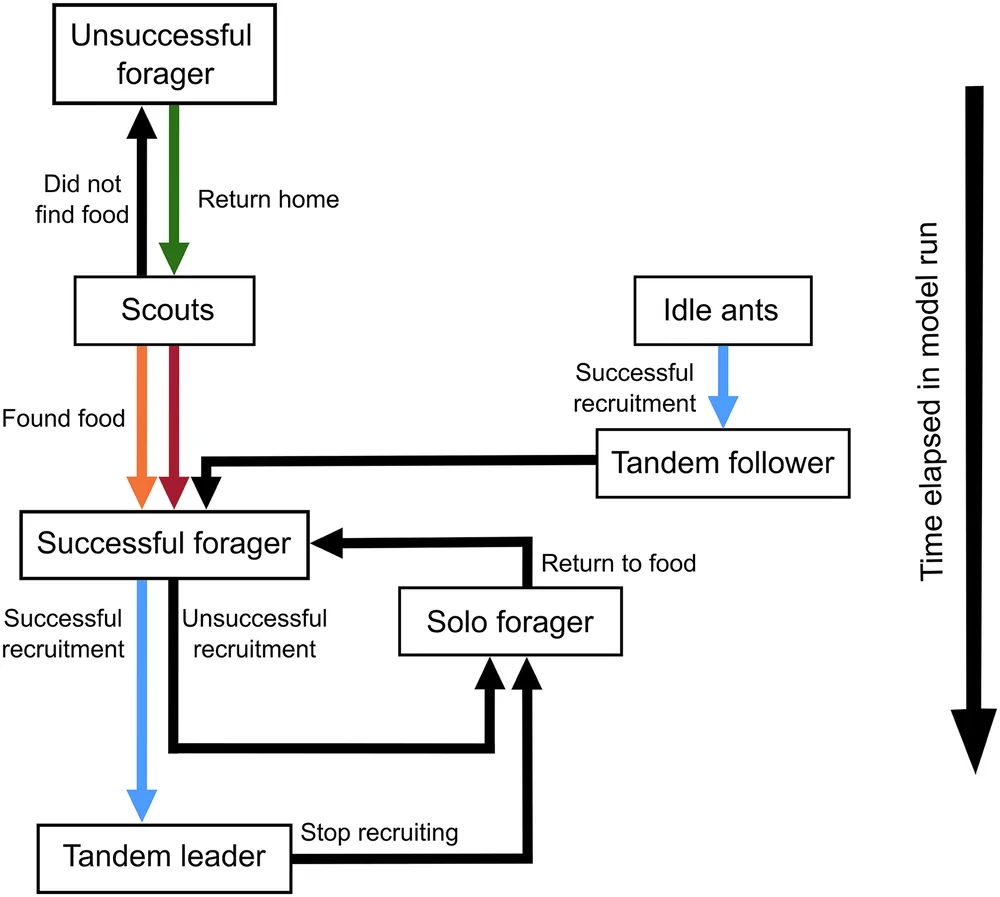
Process flowchart for agents in the model. Boxes represent different states that agents can occupy in the model, and arrows indicate transitions between state, and the conditions for transitions to occur. Colours indicate different behaviours that can be affected by infection in individuals (light green - walk speed, red - distance at which food cue is perceived, orange - turning angle, blue - probability of recruitment, dark green - giving up time).

In the absence of infection, ants are assigned to classes describing their participation in the collective foraging task (Fig. 4). At the start of each simulation, no ants have prior knowledge of the location of food, and ants are assigned as either scouts that search the arena for food using a random walk or inactive ants that wait at the nest to be recruited by a successful scout ant. Scout ants choose a random heading direction when leaving the nest, and in all subsequent time steps use a constrained random walk to search for food with walk speed 0.8 cm/s (Fig. 1b). The heading direction is drawn from a uniform distribution centred on the current heading with turning angle α_gen = 90 (i.e heading ~ U[-α_gen, α_gen]), and the ant moves 0.8 cm in the chosen direction. Once individuals are close enough to the food to perceive it (d_food = 20 cm), they move towards it in a more directed fashion, where they reorient towards the food every 5 seconds (see supplementary material) with a lower turning angle (α_food = 30) (Fig. 1c). Both α_food and d_food were chosen to reflect empirically observed food detection rates (see Supplementary Material).

Once a scout ant finds the food, she is reclassified as a successful forager that returns to the nest with food, moving in a constrained random walk while reorienting to home. Once at the nest, successful foragers can try to recruit an inactive ant to follow them back to the food with probability p_leading = 0.25. If the inactive ant accepts (with probability p_following = 0.25), they embark on a tandem run in a constrained random walk while reorienting to the food (Fig. 1e), and the successful forager is reclassified as a tandem leader, while the inactive ant becomes a tandem follower. After the tandem follower is led to the food, the tandem run disbands, and the tandem leaders and followers independently return to the nest as successful foragers where they may recruit other ants or make solo trips to the food. If a successful ant fails to recruit a tandem follower, she becomes a solo forager that walks using a food-oriented constrained random walk and is reassigned as a successful forager on her return home. If a scout ant is unable to find food after 600 time steps (an endurance threshold known as the disappointment time) (Fig. 1d), she returns home as an unsuccessful forager, and after a short delay, subsequently leaves the nest as a scout. The status of each ant in this process is visualized as a flowchart (Fig. 4).

### Ant infection

Since the goal of this model is to explore the impacts of infection on collective behaviour, and because bouts of foraging occur on relatively short timescales of ~90 min, we do not model infection transmission and instead consider infection status to be a fixed attribute of individual ants for the duration of a foraging bout. Thus, we vary the initial proportion of the ant colony infected (i.e. the infection prevalence) as a proxy for pathogen contagiousness without having to specify the transmission mode. We also ignore infection-induced mortality on this timescale. As such, our approach likely underestimates the full impacts of infection on collective foraging if social contacts such as tandem running can result in transmission, or if individuals die early in the foraging bout, reducing the potential of recruiting inactive ants to the task (see also Discussion).

When infection is present in the colony, a fixed number of ants are randomly assigned as infected according to the infection prevalence (see Supplementary). Infected ants experience sub-lethal costs of infection that manifest through altered behavioural parameters relating to locomotive performance, orientation towards food, social recruitment and endurance, all of which have been observed in collectively foraging species (Table 1). Changes in behaviour to model parameter *P* due to presence of infection are modelled as a proportional reduction in that parameter:

1 $${P}_{i}=\left(1-c\right)P$$

where *P* and *Pi* are the parameter values for uninfected and infected hosts respectively, and *c* is the cost of infection, which varies from 0 (no cost) to 1 (failure to implement a behaviour). In any simulation experiment, we only allow one host behaviour to be affected at a time; thus, all infection costs to individuals scale linearly with *c*. However, since social recruitment to foraging involves both a leader and a follower, the probability of a tandem run forming scales quadratically with the cost if both the leader and follower are infected (Fig. 1e). In the main simulations we assume that movement of a tandem pair depends on the infection status of the tandem leader, so if a leader is uninfected, the tandem pair moves as if uninfected even if the follower is an infected individual. We explored an additional model scenario where the movement performance of the tandem is reduced by infection if either the leader or follower are infected (Supplementary Figs. S3, S4).

### Simulation experiments

In our simulation experiments, we varied infection prevalence, the magnitude of the cost of infection (c), and the behavioural process affected by infection (Table 2). We performed 1000 simulation runs for each combination of these three variables and recorded the performance metrics (described below) for each run.

**Table 2:** Experimental scenarios explored in our model

What was manipulated	Scenarios	Values
Infection prevalence	Low, med, high	0.1, 0.5, 0.9
Cost of infection (*c*)	Low to high	0.1 to 1 in steps of 0.1
Behavioural processes	See Table 1	a) Individual behavioural processes were affected one at a time b) Individual behavioural processes were covaried with social costs

We carried out a sensitivity analysis to test how sensitive our results were to our baseline parameter estimates for uninfected ants, by repeating each of our simulation experiments where each baseline parameter was altered by 土20%.

### Metrics of performance - measuring impact of infection on colony performance

For each simulation run, we quantified how individual costs of infection scaled up to influence the efficiency of collective foraging. To do this, we defined the colony-level foraging performance as the net energy gain to the colony from foraging after subtracting the net energy expended during foraging. This can be expressed as

2 $${colony}.{performance}={net}.{energy}.{gain}-{net}.{energy}.{use}={b}_{{tot}}* {gain}.{per}.{trip}-\,{d}_{{tot}}* {metabolic}.{cost}$$

The net energy gain from foraging was measured by multiplying the total number of successful foraging trips, *b*_*tot*_, (i.e. each time a food item was brought back to the home nest) by the energy gained from food acquired by one ant on a successful foraging trip. Ants were estimated to lose energy based on the distance walked outside the nest while searching and/or foraging. The net energy loss was estimated by multiplying the total distance walked by all ants during a foraging bout, *d*_*tot*_, by the metabolic cost of walking 1 cm.

We quantify the impacts of infection on colony-level performance as the ratio of the performance of an infected colony to the average performance (averaged over 100 simulations) of an uninfected colony:

3 $${relative}.{performance}=\frac{{colony}.{performance}}{{{colony}.{performance}}_{U}}$$

Values closer to one indicate little to no effects on relative performance, while

values less than zero indicate failure of the foraging bout (that is, the food brought in by foraging is less than the energy expended by the colony during foraging). In order to assess how infection altered the amplification of recruitment behaviours, we recorded the number of recruitment events made in each simulation run. We averaged these performance metrics over 100 runs for each simulation experiment.

### Metrics of performance - measuring impact of infection on average individual performance

To test whether reciprocal effects of infection could lead to (i) reduction in performance of uninfected individuals through interactions with infected individuals, or (ii) improvement of average performance of infected individuals through interactions with uninfected individuals, we recorded the average energetic contribution of an infected and uninfected ant for each simulation experiment. The energetic contribution for individuals by infection status was calculated using Eq. (2) to calculate the total energetic gain by all individuals of the same infection status and then dividing by the number of individuals of that infection status. Thus, the average performance of infected individuals is given by:

4 $${E}_{{infected}}={E}_{{total}.{infected}}/{n}_{{infected}}$$

And the average performance of uninfected ants is

5 $${E}_{{uninfected}}={E}_{{total}.{uninfected}}/{n}_{{uninfected}}$$

We then took the average of these performance metrics over 100 runs for each simulation experiment. To test (i), we compared the energetic contribution made by the average uninfected individual with the average performance of an individual in a disease-free colony (average performance of an individual in the absence of infected individuals). To test (ii), we compared the energetic contribution made by the average infected individual with the average performance of an individual in a 100% infected colony (average performance of an individual in the absence of uninfected individuals).

## Supplementary information

Supplementary Information

## Data Availability

The underlying datasets generated for this study are available on Github and can be accessed via this link https://github.com/Supraja-R/abm_sublethal_effects.git.

## Code availability

The underlying NETLOGO code for this study is available in Github and can be accessed via this link https://github.com/Supraja-R/abm_sublethal_effects.git.
