# Supplementary material for: Sublethal costs of infection reduce performance of collective tasks in social insects: a modelling study
Source: Npj Entomol. 2026 Nov 5;1(1):7. doi: 10.1038/s44434-026-00007-z (PMC13739735; doi:10.1038/s44434-026-00007-z)
Supplement: Supplementary file 1 [file 44434_2026_7_MOESM1_ESM.docx]

**Supplementary Materials:**

**S1. Sensitivity of model outcomes to infection prevalence and baseline parameters**

Fig.S1: **Relative colony performance as a function of infection prevalence** (a) when individual behaviors are impacted by infection (b) when individual behaviors and social interaction costs are co-varied. Relative colony performance is the net energy gain to an infected colony (food brought to the colony minus the total energy expenditure of all ants engaged in the foraging task) normalized by the performance of an infection free colony; and the cost of infection is the proportionate reduction in an individual or social behavior due to infection. Each of these metrics is multiplied by 100 so that results are expressed as a percentage. Colors indicate which individual behaviors are impacted by infection (light green - walk speed, red - distance at which food cue is perceived, orange - turning angle, dark green - giving up time). In (b) the blue line is the cost of infection to social interactions (reduced probability of recruitment when at least one individual in the dyad is infected), and the colors of the other lines correspond to the corresponding individual cost of infection in (a). The black horizontal line represents the performance of a disease-free colony. All simulations were run at 50% cost of infection for the duration of one foraging bout (90 minutes). Plotted values represent the mean performance averaged over 1,000 simulation runs.

Fig.S2: **Number of recruitment events as a function of cost of infection.** Number of recruitment events as the cost of infection to different behavioral processes increases (a) when individual behaviours are impacted by infection (b) when individual behaviours and social interaction costs are co-varied. Cost of infection is the proportionate reduction in an individual or social behavior due to infection. Colours indicate which individual behaviours are impacted by infection (light green - walk speed, red - distance at which food cue is perceived, orange - turning angle, dark green - giving up time). In (b) the blue line is the cost of infection to social interactions (reduced probability of recruitment when at least one individual in the dyad is infected), and the colours of the other lines correspond to the corresponding individual cost of infection in (a). The black horizontal line represents the number of recruitment events in a disease-free colony. All simulations were run at 50% infection prevalence for the duration of one foraging bout (90 minutes). Plotted values represent the mean number of recruitment events averaged over 1,000 simulation runs.

Fig.S3: **Relative colony performance as a function of cost of infection increases under alternative assumptions of how infection impacts recruitment.** (a) when individual behaviors are impacted by infection (b) when individual behaviors and social interaction costs are co-varied as the cost of infection to different behavioral processes increases. Relative colony performance is the net energy gain to an infected colony (food brought to the colony minus the total energy expenditure of all ants engaged in the foraging task) normalized by the performance of an infection free colony; and the cost of infection is the proportionate reduction in an individual or social behavior due to infection. Each of these metrics is multiplied by 100 so that results are expressed as a percentage. Colors indicate which individual behaviors are impacted by infection (light green - walk speed, red - distance at which food cue is perceived, orange - turning angle, dark green - giving up time). In (b) the blue line is the cost of infection to social interactions (reduced probability of recruitment when at least one individual in the dyad is infected), and the colours of the other lines correspond to the corresponding individual cost of infection in (a). (c) The difference in relative colony performance due to social costs of infection, calculated by subtracting the performance of its colored line in (b) from the corresponding line in (a).The black horizontal line represents the performance of a disease-free colony. All simulations were run at 50% infection prevalence for the duration of one foraging bout (90 minutes). Plotted values represent the mean performance averaged over 100 simulation runs.

Fig.S4: Impacts to average individual performance under alternative assumptions of how infection impacts recruitment. Reciprocal effects of infection status on individual performance (i.e. the average energetic contribution of individuals according to their infection status) in model scenario where costs of infection to either tandem leader or tandem follower reduces performance of recruitment event. Columns indicate (left) the reduction in uninfected individuals’ performance due to infected individuals and (right) the increase in infected individuals’ performance through interactions with uninfected individuals; rows indicate (top) infection costs to individual behaviours only (indicated by arrow colour) or (bottom) infection costs to both individual and social behaviour. (a) on average, relative energetic contribution of uninfected individuals decreases with increasing infection prevalence (indicated by arrow opacity) and (b) relative energetic contribution of infected individuals increases when uninfected individuals are relatively common. Reciprocal effects of infection status on individual performance when explicitly co-varying individual costs with social costs; (c) on average, relative energetic contribution of uninfected individuals decreases with increasing infection prevalence and (d) relative energetic contribution of infected individuals increases when uninfected individuals are relatively common. Relative energetic contribution of an individual is the net energy gained by an ant (food brought minus energy expended by walking) normalized by the average energy contribution of an uninfected ant (see Methods). This metric is multiplied by 100 so that results are expressed as a percentage. Line colors represent the behaviour impacted by infection, line opacity indicates the infection prevalence, and the cost of infection to the impacted behavior is 50%. The dots at the base of each vertical arrow represent performance when all individuals have the same infection status (all uninfected in (a,c), all infected in (b,d)), and the arrowheads represent the average performance of individuals of one infection status in the presence of individuals of the opposite infection status. Plotted values represent the average individual performance over 100 simulation runs.

Fig.S5. Sensitivity of results to baseline parameters - relative colony performance as cost of infection to individual behaviour increases. Each panel represents the colony performance over a +/- 20% in our baseline parameter values for (a) walk speed (b) giving up time (c) distance at which food cue is perceived (d) turning angle (e) probability of recruitment. All simulations were run at 50% disease prevalence for the duration of one foraging bout (90 minutes). Colours indicate different behavioural mechanisms that can be affected by infection in individuals (light green - walk speed, orange - turning angle, red - distance at which food cue is perceived, dark green - giving up time), and the presence of colour in the background of the plot signify which parameter’s baseline values were varied. Line type indicates the variation in baseline parameter - solid is the baseline value, dotted lines are -20% in the baseline value and dashed lines are +20% in the baseline values

Fig.S6: Sensitivity of results to baseline parameters - relative colony performance as cost of infection to individual and social behaviour increases. Each panel represents the colony performance over a +/- 20% in our baseline parameter values for (a) walk speed (b) giving up time (c) distance at which food cue is perceived (d) turning angle (e) probability of recruitment. All simulations were run at 50% disease prevalence for the duration of one foraging bout (90 minutes). Colours indicate different behavioural mechanisms that can be affected by infection in individuals (light green - walk speed, orange - turning angle, red - distance at which food cue is perceived, blue - probability of recruitment, dark green - giving up time), and the presence of colour in the background of the plot signify which parameter’s baseline values were varied. Line type indicates the variation in baseline parameter - solid is the baseline value, dotted lines are -20% in the baseline value and dashed lines are +20% in the baseline values

**S2. Full description of agent-based model**

We outline a description of our agent-based model using the ‘Overview, Design Concepts and Details’ protocol, with some modifications to reduce redundancy ^1^.

**Purpose**

The overall purpose of the model was to explore how sublethal effects of infection to individual behaviour could scale up and affect collective outcomes in social insect colonies, using collective foraging in *Temnothorx* ants as a model organism.

**Entities, State Variables and Scales**

In all our model runs we simulated 100 active foraging ants. At the start of the model run foragers are divided into two different types, scouts (N = 25) and recruits (N = 75), based on typical proportions of scouts and recruits in social insect colonies ^2,3^. We were interested in understanding colony level impacts of sublethal changes to individual behaviour. To do this, ants were assigned an infection status at the start of the model of either “uninfected” or “infected”, and infected ants experienced a reduction in individual behaviour.

Agents were located on a two-dimensional square grid with 140 X 140 patches. Following the previous model, the width of a patch corresponded to 1 cm in our model. Agents could occupy any position in the area and were not restricted to edges of a patch. The nest was located at the centre patch and all agents started at this position. We designed the simulated colony’s foraging scenario to mimic experimental setups used to study collective foraging behaviour in these ants^4^. A single food patch was provided to the colony for foraging at a distance of 40 cm from the nest. The food patch was *ad libitum* and provided the energetic equivalent of 1 M sucrose solution when ants foraged. Simulations were run in discrete time steps, where each time step corresponded to 1 second and each simulation was run for 90 min, which is based on empirical data about the length of foraging bouts in *Temnothorax* ants^4^.

**Parameterization**

Table S1: Variables used in the agent-based model

| **Variable** | **Value** | **Information source** |
| --- | --- | --- |
| Distance from nest to food source | 40 cm | ^5^ |
| Energy gained on foraging | 0.75 J | ^5^ |
| Walk speed | 0.8 cm/sec | ^5^ |
| Food sensing distance | 20 cm | See *Ant movement* submodel |
| Orientation angle on sensing food | 30° | See *Ant movement* submodel |
| Feeding time | 120 sec | ^5^ |
| Time a returned forager stays back in the nest | 60 sec | ^5,6^ |
| Probability of leading a tandem run | 0.25 | See *Recruitment* submodel |
| Probability of following a tandem run | 0.25 | See *Recruitment* submodel |
| Energy cost of walking outside | 2.446 × 10^-7 J/tick | ^5^ |
| Time spent by scout ant searching for food before returning home | 600 | ^5^ |

**Process overview**

Individual foraging ants collected food from a provided food source and returned to the home nest to unload food. Ants could participate in foraging as scout ants that independently search for the food or on being recruited to the food by a knowledgeable individual. The amount of food brought back to the nest is equivalent to the energy gained by the colony, while the distance spent walking by ants is the energy lost by the colony.

**Submodels**

*Ant movement*

All scouting ants leaving the nest at the start of the simulation pick a random starting direction to move in. Scouts performed a random walk when moving, implemented similarly to the original model^5^. When scouts reach within the radius of their sensing distance from the food, they start performing a directed random walk, constraining their turning angle and orienting every 5 seconds towards the food. Ants that have found food can make multiple trips to and from the food or recruit other individuals. Knowledgeable ants that return to food reorient towards the food every 5 sec, as they have learned the location of the food site^4^. These ants move with a larger turning angle near the nest and walk more directly with a smaller turning angle when they are within sensing distance of the food. The values of the turning angle and sensing distance were determined by running model scenarios with a single ant across a range of turning angle and sensing distance values. Each combination was run 100 times and the proportion of runs where the food was found and number of trips made to the food were calculated and plotted (Fig S7). Values of sensing distance and turning angle that aligned with empirical observations of food detection and visitation were selected.

Fig.S7: Sensitivity of ant foraging behavior to sensing distance and turning angles. (a) Food discovery rates and (b) Number of food visits made by a single ant across 100 model scenarios with movement rules governed by a range of sensing distance and turning angle values

*Recruitment via tandem running behaviour*

Knowledgeable ants probabilistically recruit ants waiting at the home nest to the food site, and recruits probabilistically join an invitation to recruitment. These probability values were determined by running model scenarios with two ants across a range of leading and following probability values. Each combination was run 100 times and the proportion of food visits that were tandem runs were calculated and plotted (Fig S8). Probability of leading and following were set at 0.25, which led to a recruitment probability of 0.6 to a high quality food site 40 cm away, matching empirical observations made by Grüter et al., 2018 (Fig S8).

Fig.S8: Sensitivity of ant recruitment behavior to changes in individual leading and following probabilities. Recruitment rates over the whole model run in a two-ant model scenario across 100 model scenarios with recruitment rules governed by a range of leading and following probabilities.

*Ant infection status*

At the beginning of each model run, ants are randomly assigned an infection status (“uninfected” or “infected”) according to the colony’s infection prevalence. Assignment was independent of task role, such that scouts and recruits had equal probabilities of infection; however, a greater number of infected recruits was expected due to their higher abundance.

**References:**

1.	Grimm, V. *et al.* A standard protocol for describing individual-based and agent-based models. *Ecol. Model.* **198**, 115–126 (2006).

2.	Seeley, T. D. *The Wisdom of the Hive: The Social Physiology of Honey Bee Colonies*. (Harvard University Press, 2009).

3.	Shaffer, Z., Sasaki, T. & Pratt, S. C. Linear recruitment leads to allocation and flexibility in collective foraging by ants. *Anim. Behav.* **86**, 967–975 (2013).

4.	Sasaki, T., Danczak, L., Thompson, B., Morshed, T. & Pratt, S. C. Route learning during tandem running in the rock ant Temnothorax albipennis. *J. Exp. Biol.* **223**, jeb221408 (2020).

5.	Goy, N., Glaser, S. M. & Grüter, C. The adaptive value of tandem communication in ants: Insights from an agent-based model. *J. Theor. Biol.* **526**, 110762 (2021).

6.	Grüter, C., Wüst, M., Cipriano, A. P. & Nascimento, F. S. Tandem Recruitment and Foraging in the Ponerine Ant Pachycondyla harpax (Fabricius). *Neotrop. Entomol.* **47**, 742–749 (2018).
